# The “contrast agent separation” sign in a patient on veno-arterial extracorporeal membrane oxygenation undergoing computed tomography scan angiography: A case report

**DOI:** 10.1097/MD.0000000000046986

**Published:** 2026-01-09

**Authors:** Yanbo Lou, Xiaojian Jia, Jingjin Wu, Caiyou Ding

**Affiliations:** a Department of Vascular Surgery, The Fourth Affiliated Hospital of School of Medicine, and International School of Medicine, International Institutes of Medicine, Zhejiang University, Yiwu, China.

**Keywords:** aortic dissection, computed tomography angiography (CTA), contrast agent separation (CAS), transesophageal echocardiography (TEE)

## Abstract

**Rationale:**

In computed tomography angiography (CTA), the phenomenon of contrast agent separation (CAS) is one of the key imaging features for diagnosing arterial dissection. This case highlights that the “contrast agent separation” (CAS) sign in CTA after veno-arterial extracorporeal membrane oxygenation (VA-ECMO) may result from retrograde perfusion exceeding native cardiac output, causing uneven contrast mixing and potential misdiagnosis as Type A aortic dissection (AD). CTA combined with transesophageal echocardiography (TEE) is crucial for accurate diagnosis and management of such acute aortic conditions.

**Patient concerns:**

A 76-year-old man suddenly lost consciousness at home. After the arrival of the 120-emergency team, the patient was assessed to be in cardiac and respiratory arrest. Cardiopulmonary resuscitation (CPR) was initiated, followed by emergency endotracheal intubation.

**Diagnoses:**

Cardiopulmonary arrest, cardiogenic shock, iatrogenic Type B26 AD, dilated cardiomyopathy, coronary heart disease, and atrial fibrillation.

**Interventions:**

After VA-ECMO, CTA, and TEE were performed.

**Outcomes:**

The patient’s prognosis is extremely poor, the family members have made a decision to forgo further treatment.

**Lessons:**

The CAS sign in CTA after VA-ECMO may result from retrograde perfusion exceeding native cardiac output, causing uneven contrast mixing and potential misdiagnosis as Type A AD. CTA combined with TEE is crucial for accurate diagnosis and management of such acute aortic conditions.

## 1. Introduction

In computed tomography angiography (CTA), the phenomenon of contrast agent separation (CAS) is one of the key imaging features for diagnosing arterial dissection. This case highlights that the “contrast agent separation” (CAS) sign in CTA after veno-arterial extracorporeal membrane oxygenation (VA-ECMO) may result from retrograde perfusion exceeding native cardiac output, causing uneven contrast mixing and potential misdiagnosis as Type A aortic dissection (AD). CTA combined with transesophageal echocardiography (TEE) is crucial for accurate diagnosis and management of such acute aortic conditions.

## 2. Case presentation

**Figure 1.**
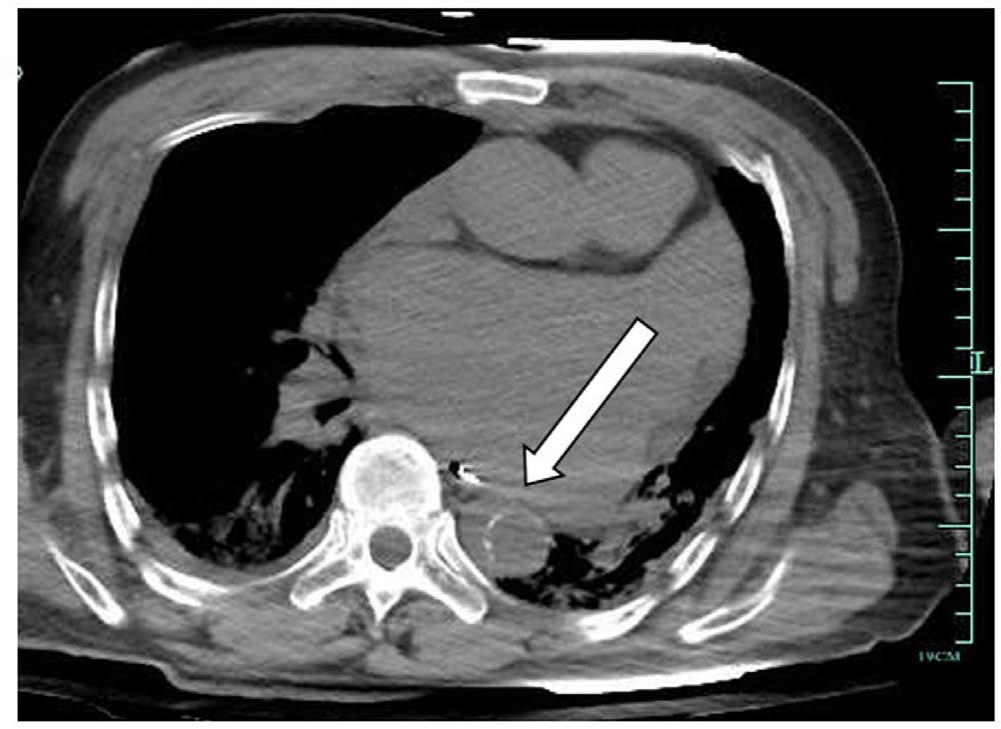
Computed tomography of the chest demonstrating displacement of the aorta calcification.

**Figure 2.**
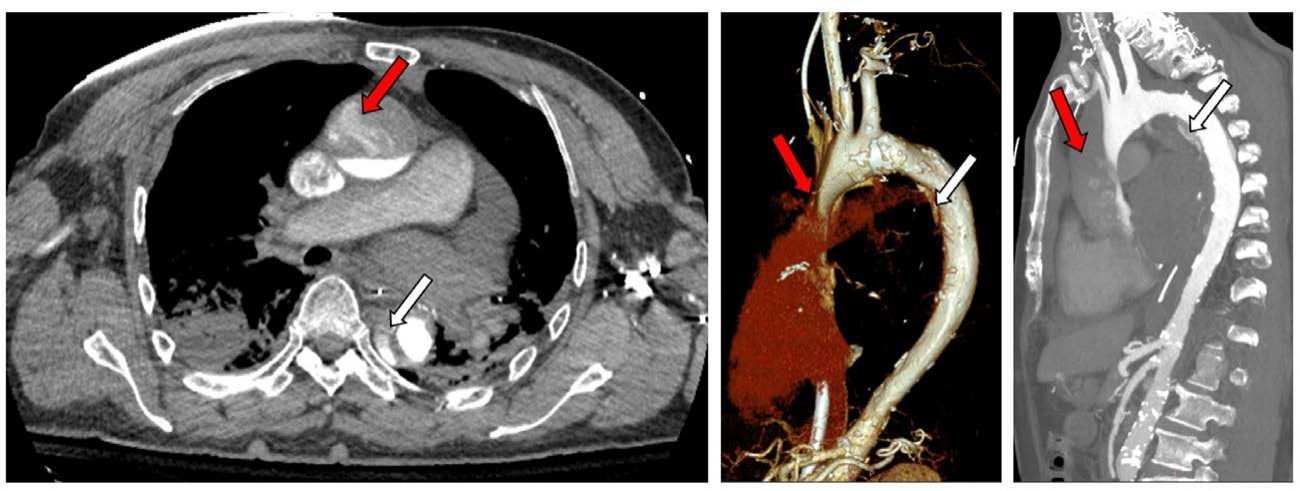
Aortic CTA showed aortic dissection without pericardial effusion. CTA = computed tomography angiography.

**Figure 3.**
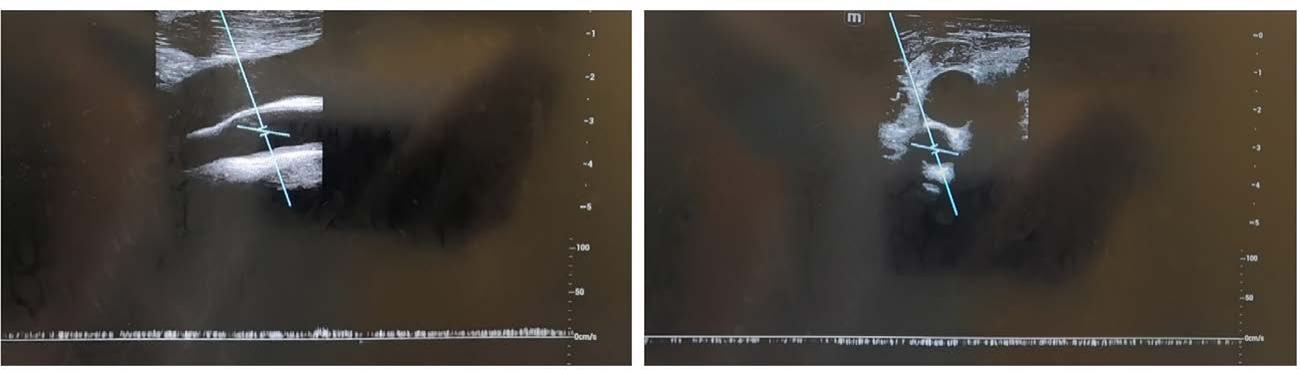
Bedside ultrasound revealed unidirectional pulseless flow in the right common carotid artery.

**Figure 4.**
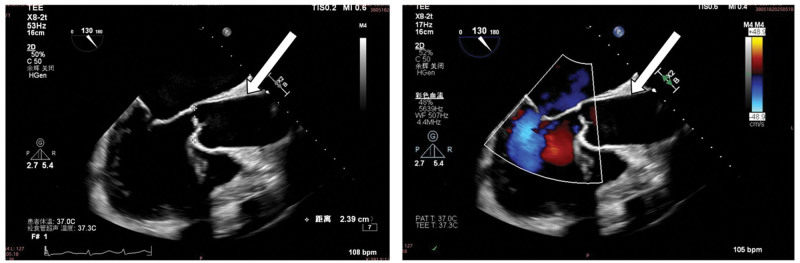
The transesophageal echocardiography shows dilation of the ascending aorta, with no evidence of dissection.

We saw the CAS sign in the following clinical context. A 76-year-old man suddenly lost consciousness at home. After the arrival of the 120-emergency team, the patient was assessed to be in cardiac and respiratory arrest. Cardiopulmonary resuscitation (CPR) was initiated, followed by emergency endotracheal intubation. The electrocardiogram revealed ventricular fibrillation, which was treated with electrical defibrillation. Upon arrival at the emergency department, the team initiated VA-ECMO using a multichannel drainage cannula (pump speed, 2600 rotations/min; flow rate, 3.0–3.5 L/min). After approximately 90 minutes of resuscitation, the patient regained spontaneous circulation and breathing. Then CTA (Figs. 1 and 2), ultrasound (Fig. 3) and TEE (Fig. 4) were performed. The CTA showed that thoracoabdominal AD with suspected mural thrombosis in the false lumen; poor opacification in the brachiocephalic trunk and ascending aorta. The patient has a history of hypertension, dilated cardiomyopathy, coronary heart disease, and atrial fibrillation.

The most likely diagnosis are cardiopulmonary arrest, cardiogenic shock, iatrogenic Type B26 AD, dilated cardiomyopathy, coronary heart disease, and atrial fibrillation. Aortic dissection is considered to be iatrogenic Type B AD. In the patient’s CTA imaging, the ascending aorta shows a layered distribution of contrast agent, appearing horizontally stratified. Even after adjusting the window width and level, no intimal flap or tear was identified. In this case, the patient underwent retrograde perfusion via the left femoral artery for VA-ECMO support therapy. This finding may represent a false appearance resulting from retrograde perfusion pressure and flow from VA-ECMO exceeding the patient’s native cardiac output, which prevents uniform mixing of the contrast agent. This phenomenon can easily be misdiagnosed as a Type A AD. Bedside ultrasound showed monophasic, pulseless blood flow in the right common carotid artery. The TEE confirmed the integrity of the ascending aortic lumen, further supporting the above conclusion. Unfortunately, the patient’s prognosis is extremely poor, the family members have made a decision to forgo further treatment.

## 3. Discussion

High-flow retrograde perfusion of ECMO may impede the uniform mixing of contrast medium, resulting in a stratified appearance similar to that of AD.^[1,2]^ The first report of false-positive diagnosis of AD associated with femoral cardiopulmonary bypass by TEE in 1998.^[3]^ CTA is the defacto test of choice to diagnose AD, but non-electrocardiogram-gated CTA has potential false positivity due to premature acquisition and poor dye mixing due to low-flow (low cardiac output and/or shock states) resulting in differential flow and/or dye streaming in the ascending aorta and gravitational dispersion of the dye posteriorly.^[4]^

A similar case reports where the initial CTA misdiagnosed the condition as AD,^[1]^ and the diagnosis was later corrected to left ventricular rupture through a second CTA and echocardiography. This suggests that caution is required when interpreting vascular imaging in ECMO patients.

In this case, TEE confirmed the integrity of the ascending aortic lumen and ruled out true dissection. Literature emphasizes that TEE can identify intimal flaps (such as the pseudo-intimal flap not found in this case) and assess aortic integrity in ECMO patients.^[5,6]^

Bedside ultrasound showed monophasic pulseless blood flow in the right common carotid artery, which is consistent with the nonpulsatile perfusion characteristic caused by VA-ECMO,^[7,8]^ further supporting hemodynamic changes rather than anatomical lesions.

Aoki et al clearly reports that adjustment of VA-ECMO cannulation position may lead to AD.^[9]^ However, no intimal tear was found in this case, which is more consistent with hemodynamic artifacts. Recent research points out that VA-ECMO increases left ventricular afterload, leading to elevated aortic pressure and changes in end-diastolic pressure, which may exacerbate the abnormal distribution of contrast medium.^[10]^ It was recommended to combine CTA, ultrasound, and TEE to reduce misdiagnosis.^[1,2,7]^ In this case, the diagnosis was corrected by TEE, highlighting the necessity of dynamic monitoring. Literature suggests that the interaction between ECMO and the native circulation should be monitored.^[8,10]^

Therefore, CTA plus TTE is the best combination for diagnosing AD and its complications, and allows important morphological and dynamic aspects of AD to be assessed and appropriately managed.^[11]^ Multimodality imaging adds key information about urgency indicators and the associated complications. A correct and high-quality diagnostic work-up improves the poor prognosis in this emergency condition.^[12]^ Also, we can relate the findings to broader thromboembolic and hemodynamic risk assessment strategies, CHA2DS2-VASc score and CLOTS-AF score in patients offer complementary insights into cardiovascular risk stratification relevant to this case’s clinical context.^[13,14]^

## 4. Conclusion

Aortic dissection is an acute and critical condition. CTA plus TTE is pivotal in establishing the diagnosis and guiding management. The integration of multimodal imaging in AD diagnosis represents a critical advancement in emergency cardiovascular care, addressing the limitations of individual modalities while optimizing diagnostic accuracy and therapeutic decision-making. Early recognition may help avoid complications and improve patient outcomes.

## Acknowledgments

We would like to credit the patient for her participation in this case study.

## Author contributions

**Data curation:** Jingjin Wu, Caiyou Ding.

**Writing – original draft:** Xiaojian Jia.

**Writing – review & editing:** Yanbo Lou.
